# Supplementary material for: Parallel multi-criteria decision analysis for sub-national prioritization of zoonoses and animal diseases in Africa: The case of Cameroon
Source: PLoS One. 2024 Jun 25;19(6):e0295742. doi: 10.1371/journal.pone.0295742 (PMC11198839; doi:10.1371/journal.pone.0295742)
Supplement: S1 Fig — (PDF) [file pone.0295742.s001.pdf]

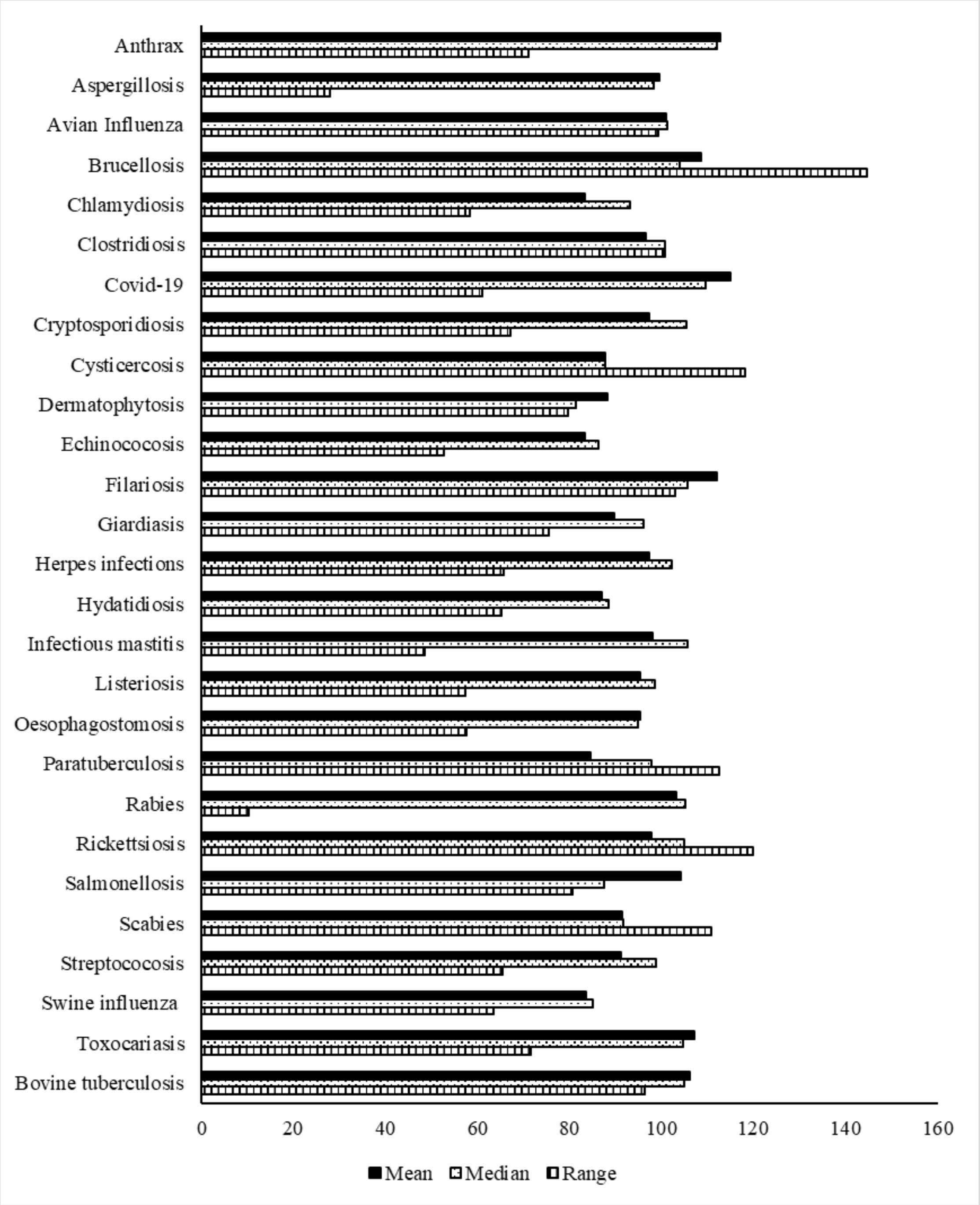

S1 Fig. Graph showing the mean, median scores and the range of the scores among the experts per zoonosis.
