# Supplementary material for: Parallel multi-criteria decision analysis for sub-national prioritization of zoonoses and animal diseases in Africa: The case of Cameroon
Source: PLoS One. 2024 Jun 25;19(6):e0295742. doi: 10.1371/journal.pone.0295742 (PMC11198839; doi:10.1371/journal.pone.0295742)
Supplement: S1 Table — (PDF) [file pone.0295742.s003.pdf]

S1 Table. Criteria experts involved in the prioritization of zoonoses

N	Gender	Institutions	Position/Responsability	Area of expertise
1.	M	Ministry of forest and wildlife	Forest engineer	Wildlife
2.	M	Ministry of livestock, fisheries and animal industries	Minister Advisor N°1, Former chief veterinary officer	Pathology
3.	M	Economic Community of Central African States	One Health specialist, REDISSE IV project	Epidemiology
4.	M	Ministry of livestock, fisheries and animal industries	Permanent secretary of the Animal Disease Surveillance Network	Epidemiology
5.	M	National Veterinary Laboratory	Head of animal pathology service	Virology
6.	M	Private veterinary practitionner « Equavet »	Chair of Epidemiology and Public Association, Head of the communication unit of the National veterinary counsil	Epidemiology, One Health
7.	F	Ministry of public Health	Deputy permanent secretary of the national programme for control and prevention of emerging and reemerging zoonoses, health manager	Public health, emergency management

S1 Table. Continued

N	Gender	Institutions	Position/Responsability	Area of expertise
8.	M	Programme Zoonose	Permanent secretary of the national programme for control and prevention of emerging and reemerging zoonoses	Epidemiology
9.	M	Ministry of livestock, fisheries and animal industries	Deputy director of the veterinary inspection and public health Unit	Bacteriology
10.	M	Ministry of forest and wildlife	Forest engineer	Wildlife
11.	F	University of Yaounde, Ministry of public health	Minister Advisor N°2, lecturer and researcher	Parasitology
12.	M	Centre Pasteur du Cameroun	Focal point One Health, researcher	Virology
13.	F	Ministry of public Health	Epidemiologist at the direction of epidemic and pandemic disease control and prevention unit	Epidemiology
14.	M	University of Bamenda	Division chief at the College of technologies, lecturer and reseacher	Microbiology, epidemiology
15.	M	Univerté des Montagnes, AFROHUN	Country manager of Afrohun, lecturer and researcher	Epidemiology,wildlife
