# Supplementary material for: Parallel multi-criteria decision analysis for sub-national prioritization of zoonoses and animal diseases in Africa: The case of Cameroon
Source: PLoS One. 2024 Jun 25;19(6):e0295742. doi: 10.1371/journal.pone.0295742 (PMC11198839; doi:10.1371/journal.pone.0295742)
Supplement: S2 Table — (PDF) [file pone.0295742.s004.pdf]

S2 Table. Criteria experts involved in the prioritization of animal diseases

N	Gender	Institutions	Position/ Responsability	Area of expertise
1.	M	EQUAVET	Private veterinarian, Chair and President of Epidemiology and Public Health Association	Epidemiology, animal health
2.	M	University of Dschang	Veterinarian, lecturer and researcher, Secretary general of the higher technical teacher training college	Microbiology
3.	M	MERIAL	Private veterinarian, MERIAL representative for Central Africa (Retired)	Pathology
4.	M	Ministry of livestock, fisheries, and animal industries	Veterinarian, former chief veterinary officer, Former director of statistic and cooperation (Retired)	Epidemiology, disease control
5.	F	Ministry of livestock, fisheries, and animal industries	Veterinarian, divisional delegate of the ministry of livestock, fisheries, and animal industries	Animal disease control
6.	M	National Veterinary Laboratory - Garou	Director of animal pathology division	Pathology
7.	M	National Veterinary Laboratory - Garou	Deputy director of animal pathology division	Entomology, virology
8.	M	National Veterinary Laboratory - Garou	Director of the vaccine production division	Bacteriology
9.	M	Ministry of livestock, fisheries, and animal industries	Regional delegate of Adamawa and Board Chairman of the National Veterinary Laboratory	Animal disease control

S2 Table. Continued

N	Gender	Institutions	Position/ Responsability	Area of expertise
10.	M	University of Ngaoundere	Deputy director, lecturer and researcher at the School of veterinary Medicine and Sciences	Microbiology
11.	M	Economic Commission of Central Africa States	One Health specialist for REDISSE IV- Project	Virology
12.	M	Ministry of livestock, fisheries, and animal industries	Former chief veterinary officer, and Minster advisor N°1	Pathology
13.	M	Ministry of livestock, fisheries, and animal industries	Deputy director and permanent secretary of the animal disease surveillance network	Epidemiology, animal disease control
14.	M	Infectious disaese detecetion and surveillance, USAID	Contry team manager, and researcher	Epidemiology,microbiology, pharmacology
15.	M	University of Douala	Lecturer and researcher	Microbiology
16.	M	Ministry of livestock, fisheries, and animal industries	Deputy director food inspection and and veterinary public health	Animal diseases control and zoonoses
17.	M	FAO, Ministry of livestock, fisheries, and animal industries (Past)	Coordinator of the Emerging Pandemic Threats II (EPT II) project, Former chief veterinary officer	Epidemiology and animal disease control
18.	M	Ministry of livestock, fisheries, and animal industries	Head of animal epiodemiosurveillance service	Epidemiology
19.	M	University of Dschang	Lecturer and researcher Faculty of agriculture and agricultural sciences (retired)	Microbiology
20.	M	Ministry of livestock, fisheries, and animal industries	Regional delegate for the center	Animal disease control
21.	M	Montagnes University and Afrohun	Lecturer, researcher, Contry manager Afrohun	Epidemiology, Wildlife
