# Supplementary material for: Parallel multi-criteria decision analysis for sub-national prioritization of zoonoses and animal diseases in Africa: The case of Cameroon
Source: PLoS One. 2024 Jun 25;19(6):e0295742. doi: 10.1371/journal.pone.0295742 (PMC11198839; doi:10.1371/journal.pone.0295742)
Supplement: S3 Table — (PDF) [file pone.0295742.s005.pdf]

S3 Table. Disease experts involved in the prioritization of zoonoses

N	Gender	Institutions	Position/Responsability	Field of expertise	Disease
1.	M	National Veterinary Laboratory - Garoua	Deputy director of animal pathology division	Virology	Anthrax, brucellosis, cryptosporidiosis, herpes infections, infectious mastitis, rabies
2.	M	National Veterinary Laboratory - Garoua	Staff	Virology	Anthrax, filariasis, rickettsiosis, salmonellosis, toxocariasis, tuberculosis
3.	M	National Veterinary Laboratory - Garoua	Staff	Virology	Avian influenza, echinococcosis, listeriosis, swine influenza
4.	M	Ministry of livestock, fisheries, and animal industries	Divisional delegate of the ministry of livestock, fisheries, and animal industries of Benoue	Animal disease control	Rickettsiosis, giardiasis, streptococosis, infectious mastitis
5.	M	Ministry of livestock, fisheries, and animal industries	Sub divisional delegate of the ministry of livestock, fisheries, and animal industries of Pitoa	Animal disease control	Anthrax, paratuberculosis, salmonellosis, streptococosis
6.	M	Ministry of livestock, fisheries, and animal industries	Sub divisional delegate of the ministry of livestock, fisheries, and animal industries of Hourna	Animal disease control	Echinococcosis, cysticercosis, rickettsiosis, swine influenza, toxocariasis
7.	M	Centre Pasteur (annexe Garoua)	Laboratory chairman	Microbiology	Bovine tuberculosis, scabies
8.	M	Cabinet Véterinaire du Pole	Private veterinarian, Director general	Animal disease control	Avian influenza, echinococcosis, giardiasis, hydatidosis, listeriosis, oesophagostomosis
9.	M	Sep Vet	Private veterinarian, Director general	Animal disease control	Clostridiosis, infectious mastitis, scabies, swine influenza

S3 Table. Continued

N	Gender	Institutions	Position/Responsability	Field of expertise	Disease
10.	M	Ministry of scientific research and innovation	Researcher at the Institute of Agricultural Research for Development- Wakwa	Microbiology	Brucellosis, cryptosporidiosis, tuberculosis, cysticercosis, hydatidosis
11.	M	National Veterinary Laboratory - Garoua	Head of the production section	Microbiology	Ascariasis, aspergillosis, clostridiosis, infectious mastitis, oesophagostomosis
12.	M	Ministry of livestock, fisheries, and animal industries	Sub divisional delegate of the ministry of livestock, fisheries, and animal industries of Bacheo	Animal disease control	Clostridiosis, cysticercosis, echinococosis, giardiasis, swine influenza .
13.	M	Ministry of livestock, fisheries, and animal industries	Regional chief of veterinary services	Animal disease control	Aspergillosis, cryptosporidiosis, filariasis, hydatidiosis, toxocariasis
14.	F	Ministry of livestock, fisheries, and animal industries	Staff at the Sub divisional delegation of the ministry of livestock, fisheries, and animal industries of Garoua I	Animal disease control	Avian influenza, aspergillosis, oesophagostomosis, paratuberculosis
15.	F	Ministry of Public Health	Staff at the integrated health - center of Beka	Public health	Aspergillosis, giardiosis, salmonellosis
16.	F	Ministry of livestock, fisheries, and animal industries	Staff at the Regional delegation of the ministry of livestock, fisheries, and animal industries for the North	Animal disease control	Dermatosis, herpes infections, paratuberculosis, streptococosis, tuberculosis

S3 Table. Continued

N	Gender	Institutions	Position/Responsability	Field of expertise	Disease
17.	M	Ministry of livestock, fisheries, and animal industries	Staff at the Sub divisional delegation of the ministry of livestock, fisheries, and animal industries of Garoua II	Animal disease control	Brucellosis, chlamydiosis, covid-19, filariasis, rabies, scabies
18.	F	Ministry of Public Health	Staff of the integrated health - center of Beka	Public health	Chlamydiosis, Covid-19, rabies, scabies
19.	F	Ministry of livestock, fisheries, and animal industries	Sub divisional delegate of the ministry of livestock, fisheries, and animal industries of Garoua II	Animal disease control	Clostridiosis, cryptosporidiosis, dermatosis, oesophagostomosis
20.	M	Ministry of forest and and wildlife	Staff of the zoological garden of of Benoué	Wildlife	Dermatosis, herpes infections,
21.	M	Ministry of public health	Chair of the integrated health - center of Ngaoundere	Public health	Chlamydiosis, dermatosis, rabies, rickettsiosis
22.	M	Ministry of scientific research and innovation	Researcher at the Institute of Agricultural Research for Development- Wakwa	Microbiology	Chlamydiosis, Covid-19, filariaris, listeriosis, paratuberculosis
23.	M	Infectious Disease detection and surveillance (IDDS-USAID)	Country team manager	Microbiolgy, epidemiology	Anthrax, avian Influenza, brucellosis, Covid-19, herpes infections, toxocarosis
24.	M	CAPHAVET, Ngaoundere agency	Private veterinarian, chair of the Ngaoundere agency	Animal disease control	Cysticercosis, hydatidosis, listeriosis, salmonellosis
25.	M	Pole Vet practice	Staff at the private vet practice	Animal disease control	Cryptosporidiosis, filariosis, listeriosis, paratuberculosis, streptococosis
