# Supplementary material for: Parallel multi-criteria decision analysis for sub-national prioritization of zoonoses and animal diseases in Africa: The case of Cameroon
Source: PLoS One. 2024 Jun 25;19(6):e0295742. doi: 10.1371/journal.pone.0295742 (PMC11198839; doi:10.1371/journal.pone.0295742)
Supplement: S4 Table — (PDF) [file pone.0295742.s006.pdf]

S4 Table. Disease experts involved in the prioritization of animal diseases

N°	Gender	Institutions	Position/ Responsability	Area of expertise	Disease
1.	M	National Veterinary Laboratory - Garoua	Deputy director of animal pathology division	Entomology, virology	Dermatophilosis, hokovirosis, Rift valley fever, trypanosomiasis
2.	M	National Veterinary Laboratory - Garoua	Researcher, staff	Virology	ASF, Covid-19, HPAI, infectious bursal disease
3.	M	National Veterinary Laboratory - Garoua	Researcher, staff	Virology	Anthrax, Covid-19, HPAI, Rift valley fever
4.	M	National Veterinary Laboratory - Garoua	Director of animal pathology division	Pathology	FMD, HPAI, lumpy skin disease, Rift valley fever
5.	M	Ministry of livestock, fisheries, and animal industries	Chief of the zoo technical and veterinary center of Garoua I	Animal disease control	Cysticercosis, lumpy skin disease, monieziasis, New Castle disease
6.	M	Ministry of livestock, fisheries, and animal industries	Sub divisional delegate of the ministry [illegible] livestock, fisheries, and animal industries Mayo-Hourna	Animal disease control	CCPP, nematodiasis, scabies, tuberculosis
7.	M	Ministry of livestock, fisheries, and animal industries	Sub divisional delegate of the ministry [illegible] livestock, fisheries, and animal industries Garoua I	Animal disease control	Babesiosis, paramphistomosis, PPR, scabies
8.	M	National Veterinary Laboratory - Garoua	Deputy head of service of bacteriology	Bacteriology	Colibaccillosis, Covid-19, pasteurellosis, HPAI
9.	M	National Veterinary Laboratory - Garoua	Head of the serology section/Division of animal pathology	Bacteriology	FMD, hokovirosis, HPAI, monieziasis/taeniasis,
10.	M	Ministry of livestock, fisheries, and animal industries	Sub divisional delegate of the ministry [illegible] livestock, fisheries, and animal industries Garoua II	Animal disease control	Anaplamosis, cowdriosis, salmonellosis, Scabies

S4 Table. Continued

N°	Gender	Institutions	Position/ Responsability	Area of expertise	Disease
11.	M	National Veterinary Laboratory - Garoua	Quality officer- HSE LANAVET	Bactériology and laboratory quality management	Campylobacteriosis, LPAI, Q-fever, viral hepatitis E
12.	M	Ministry of livestock, fisherie animal industries	Sub divisional delegate of the ministry of livesto fisheries, and animal industries of Bacheo	Animal disease control	Coccidiosis, cowdriosis, lumpy skin disease, nematodiasis
13.	M	Ministry of livestock, fisherie animal industries	Regional chief of veterinary services	Animal disease contro	CBPP, coccidiosis, PPR, tuberculosis
14.	F	Ministry of livestock, fisheries, and animal industries	Staff at the sub divisional delegate of the ministry of livestock, fisheries, and animal industries of Garoua II	Animal disease control	ASF, CCPP, Paramphistomosis, Scabies
15.	M	Ministry of livestock, fisheries, and animal industries	Chief of the zootechnic and veterinary center of Badjouma	Animal disease control	ASF, BVD, colibaccilosis, New Castle disease
16.	F	National Veterinary Laboratory - Garoua	Deputy director vaccine production division	Microbiology	CBPP, Covid-19, infectious bursal disease, Q fever
17.	M	Ministry of livestock, fisheries, and animal industries	Staff at the sub divisional delegate of the ministry of livestock, fisheries, and animal industries of Garoua I	Animal disease control	BVD, Campylobacteriosis, Viral hepatitis E, Monieziasis/taeniasis
18.	M	National Veterinary Laboratory - Garoua	Head of the production section/Division vaccine production	Bacteriology	Anthrax, CCPP, New Castle disease, pasteurellosis
19.	F	Ministry of livestock, fisheries, and animal industries	Staff at the sub divisional delegate of the ministry of livestock, fisheries, and animal industries of Garoua I	Animal disease control	Anthrax, fasciolosis, nematodiasis, tuberculosis

S4 Table. Continued

N°	Gender	Institutions	Position/ Responsability	Area of expertise	Disease
20.	F	Ministry of livestock, fisheries, and animal industries	Staff at the sub divisional delegate of the ministry of livestock, fisheries, and animal industries of Garoua II	Animal disease control	Cysticercosis, fasciolosis, heart water, viral hepatitis E
21.	M	Ministry of livestock, fisheries, and animal industries	Regional delegate of the ministry of livestock, fisheries, and animal industries of littoral	Animal disease control	CBPP, FMD, pasteurellosis, trypanosomias
22.	M	Ministry of livestock, fisheries, and animal industries	Veterinary station Wakwa	Animal disease control	Babesiosis, brucellosis, FMD, nematodiasis
23.	M	Continental Vet	Director general of the private veterinary practice	Animal disease control	BVD, LPAI, PPR, Q fever
24.	M	Institute of Agricultural Researc Development- Wakwa	Researcher	Microbiology	Brucellosis, classical swine fever, infectious mastitis, leptospirosis
25.	M	Institute of Agricultural Researc Development- Wakwa	Researcher	Microbiology	BVD, salmonellosis, leptospirosis, viral hepatitis E
26.	M	Institute of Agricultural Researc Development- Wakwa	Chief of the animal health section	Animal disease control	Babesiosis, colibaccilosis, dermatophilosis, infectious endometritis,
27.	M	Pole Vet practice	Private veterinarian, Director general	Animal disease control	Campylobacteriosis heart water, infectious bursal disease, Pasteurellosis
28.	M	AFRICAVET	Staff at the private vet practice	Animal disease control	Brucellosis, classical swine fever, leptospirosis, trypanosomosis
29.	M	AFRICAVET	Director general of the private veterinary practice	Animal disease control	Brucellosis, dermatophilosis, fasciolosis, tuberculosis

S4 Table. Continued

N°	Gender	Institutions	Position/ Responsability	Area of expertise	Disease
30.	**M**	**National Veterinary Laboratory - Garoua**	**Researcher, head of the production vaccine production division**	**Microbiology**	**IBR, LPAI, paramphistomosis, PPR**
31.	M	Ministry of livestock, fisheries, and animal industries	Chief of the zoo technical and veterinary center of Ngaoundere I	Animal disease control	Coccidiosis, IBR, infectious endometritis, salmonellosis,
32.	M	CEVA	Staff	Vet drug dispensation	infectious endometritis, infectious mastitis, lumpy skin disease,
33.	M	Ministry of livestock, fisheries, and animal industries	Training officer for the Glossina Surveillance and Eradication Mission	Animal disease control	Rift valley fever Anaplasmosis, babesiosis, trypanosomosis,
34.	M	Ministry of livestock, fisheries, and animal industries	Staff at the divisional delegation of the ministry of livestock, fisheries, and animal industries of Vina	Animal disease control	anaplasmosis, Classical swine fever, coccidiosis, colibaccilosis, New Castle disease,
35.	F	Ministry of livestock, fisheries, and animal industries	Staff at the divisional delegation of the ministry of livestock, fisheries, and animal industries of Vina	Animal disease control	infectious bursal disease, leptospirosis, monieziasis/taeniasi,
36.	M	School of veterinary Medicine and Sciences, University of Ngaoundere	Head of genetics and biostatics department, Lecturer and researcher	Animal health and genetics	African swine fever, anthrax, fasciolosis, hokovirosis
37.	M	CEVA	Delegate for the North Region	Vet drug dispensation, animal diease control	Cysticercosis, IBR, infectious mastitis, Paramphistomosis
38.	M	Ministry of livestock, fisheries, and animal industries	Director of the zoo technical center of Wakwa	Animal production and animal health	CBPP, cysticercosis, IBR, Q-fever
39.	F	Ministry of livestock, fisheries, and animal industries	Regional delegate of the North	Animal disease control	Campylobacteriosis, classical swine fever, hokovirosis, infectious endometritis
40.	M	Ministry of livestock, fisheries, and animal industries	Divisional delegate of Vina	Animal disease control	Anaplamosis, classical swine fever, dermatophilosis, infectious mastitis
