# Supplementary material for: Parallel multi-criteria decision analysis for sub-national prioritization of zoonoses and animal diseases in Africa: The case of Cameroon
Source: PLoS One. 2024 Jun 25;19(6):e0295742. doi: 10.1371/journal.pone.0295742 (PMC11198839; doi:10.1371/journal.pone.0295742)
Supplement: S5 Table — (PDF) [file pone.0295742.s007.pdf]

**S5 Table. Ranking and two-step cluster analysis of 36 zoonoses according to the completed and reduced expert models**

| | | | Reduced model | | | | | | | |
|---|---|---|---|---|---|---|---|---|---|---|
| | Complete model | | Expert group 1 | | Expert group 2 | | Expert group 3 | | Expert group 4 | |
| Zoonoses | Mean score | Rank | Mean score | Rank | Mean score | Rank | Mean score | Rank | Mean score | Rank |
| Anthrax | 112.86 | 2 | 124.45 | 2 | 121.81 | 2 | 100.75 | 7* | 104.41 | 11* |
| Aspergillosis | 99.43 | 10 | 94.40 | 15* | 98.37 | 12 | 101.28 | 6* | 103.68 | 13* |
| Brucellosis | 108.62 | 4 | 120.28 | 3 | 131.16 | 1* | 100.07 | 9* | 82.96 | 26* |
| Chlamydiosis | 83.38 | 27 | 76.85 | 26 | 83.32 | 23* | 76.93 | 23* | 96.30 | 16* |
| Clostridiosis | 96.57 | 15 | 114.79 | 5* | 99.46 | 11 | 81.20 | 21* | 90.84 | 20* |
| COVID-19 | 114.88 | 1 | 128.80 | 1 | 108.43 | 78* | 97.40 | 13* | 124.88 | 2 |
| Cryptosporidiosis | 97.40 | 13 | 90.95 | 17* | 111.25 | 5* | 98.50 | 12 | 88.90 | 21* |
| Cysticercosis | 87.65 | 22 | 82.72 | 23 | 92.55 | 17* | 67.99 | 27* | 107.34 | 8* |
| Dermatosis | 88.31 | 21 | 99.24 | 13* | 92.57 | 16* | 72.68 | 26* | 88.76 | 22* |
| Echinococosis | 83.44 | 26 | 85.12 | 20* | 75.57 | 25 | 93.15 | 16* | 79.91 | 27 |
| Filariosis | 111.99 | 3 | 111.99 | 6* | 111.98 | 4 | 116.29 | 2 | 127.01 | 1 |
| Giardiasis | 89.58 | 20 | 79.186 | 25* | 104.33 | 9* | 80.97 | 22 | 93.85 | 19 |
| Herpes infections | 97.36 | 14 | 92.17 | 16* | 109.93 | 6* | 99.32 | 11 | 88.03 | 23* |
| High pathogenic Avian influenza | 100.91 | 9 | 101.98 | 11 | 117.56 | 3* | 99.64 | 10 | 84.47 | 25* |
| Hydatosis | 86.96 | 23 | 84.62 | 21 | 88.27 | 20* | 76.63 | 24 | 98.33 | 15* |
| Infectious mastitis | 97.94 | 11 | 95.29 | 14* | 92.46 | 18* | 95.44 | 14* | 108.59 | 5* |
| Listeriosis | 95.38 | 16 | 86.88 | 18* | 88.13 | 21* | 100.55 | 8* | 105.97 | 9* |
| Oesophagostomosis | 95.35 | 17 | 85.62 | 19* | 104.82 | 8* | 94.98 | 15 | 95.98 | 17 |
| Paratuberculosis | 84.44 | 24 | 70.15 | 27 | 72.07 | 27* | 87.85 | 18* | 107.69 | 7* |
| Rabies | 103.24 | 8 | 102.41 | 10 | 102.89 | 10 | 105.80 | 5* | 101.88 | 14* |
| Rickettsiosis | 97.85 | 12 | 101.71 | 12 | 80.22 | 24* | 89.30 | 17* | 120.16 | 3* |
| Salmonellosis | 104.13 | 7 | 110.94 | 7 | 84.08 | 22* | 113.13 | 3* | 108.35 | 6 |
| Scabies | 91.46 | 18 | 110.01 | 8* | 95.83 | 13* | 73.08 | 25* | 86.93 | 24* |
| Streptococosis | 91.22 | 19 | 82.79 | 22* | 94.70 | 14* | 82.79 | 20 | 104.62 | 10* |
| Swine influenza | 83.45 | 25 | 79.98 | 24* | 73.38 | 26 | 85.88 | 19* | 94.57 | 18* |
| Toxocariasis | 107.17 | 5 | 118.26 | 4 | 94.39 | 15* | 107.33 | 4 | 108.69 | 4 |
| Tuberculosis | 106.05 | 6 | 108.98 | 9* | 89.63 | 19* | 121.71 | 1* | 103.88 | 12* |
| Bivariate Spearman rank correlation between the complete model and reduced models (Rho correlation coefficient) | | | 0.86 | | 0.66 | | 0.74 | | 0.44 | |
| *p* | | | <0.05 | | p<0.05 | | p<0.05 | | p<0.05 | |

*Ranking changed by three positions or more.
