# Supplementary material for: Parallel multi-criteria decision analysis for sub-national prioritization of zoonoses and animal diseases in Africa: The case of Cameroon
Source: PLoS One. 2024 Jun 25;19(6):e0295742. doi: 10.1371/journal.pone.0295742 (PMC11198839; doi:10.1371/journal.pone.0295742)
Supplement: S6 Table — (PDF) [file pone.0295742.s008.pdf]

S6 Table. Ranking and two-step cluster analysis of 40 animal diseases according to the completed and reduced expert models

	Complete model		Reduced model							
			Expert group 1		Expert group 2		Expert group 3		Expert group 4	
Maladies	Mean score	Rank	Mean score	Rank	Mean score	Rank	Mean score	Rank	Mean score	Rank
Anaplamosis	124.16	10	101.47	29*	134.92	5*	122.81	13*	137.45	2*
Anthrax	117.52	16	121.28	11*	116.33	21*	108.19	26*	124.30	13*
ASF	94.05	36	84.51	38	102.94	32*	106.40	27*	82.35	38
Babesiosis	99.87	34	107.38	25*	87.18	38*	113.10	22*	91.82	35
Brucellosis	134.95	1	133.56	3	141.39	1	128.13	9*	136.73	3
BVD	90.87	38	95.70	34*	103.81	27*	89.96	35*	74.00	40
Campylobacteriosis	113.15	22	116.44	15*	110.18	23	124.78	12*	101.18	28*
CBPP	107.77	26	107.29	26	104.35	26	98.87	32*	120.59	16*
CCPP	89.01	39	84.75	37	95.42	37	92.79	34*	83.09	37
Coccidiosis	100.09	33	91.87	35	115.54	22*	100.49	31	92.45	34
Colibaccilosis	116.87	17	100.26	30*	132.72	8*	117.74	17	116.78	19
COVID-19	119.72	13	124.33	10*	116.58	20*	132.52	5*	105.45	24*
CSF	101.91	32	97.19	33	103.80	28*	88.38	37*	118.26	18*
Cysticercosis	103.87	31	98.27	32	101.94	33	102.55	29	112.70	20*
Dermatophylosis	125.75	8	127.84	8	116.97	19*	137.09	4*	121.09	15*
Fasciolosis	116.23	18	118.34	12*	103.25	29*	117.59	18	125.74	11*
FMD	132.10	2	132.15	4	129.15	11*	128.55	8*	138.56	1
Heart water	91.83	37	88.62	36	99.87	34*	82.80	39	96.03	33*
Hokovirosis	106.14	28	81.72	39*	86.49	39*	131.07	7*	125.27	12*
HPAI	124.63	9	126.55	9	131.54	9	138.13	3*	102.29	27*
IBD	104.48	29	98.74	31	119.00	17*	110.27	25*	89.91	36*
IBR	106.94	27	108.27	24*	105.24	25	103.84	28	110.41	22*
Infectious endometritis	70.77	40	69.35	40	80.12	40	59.47	40	74.15	39
Infectious mastitis	104.48	30	104.15	27*	98.65	36*	114.21	20*	100.90	29
Leptospirosis	114.75	19	111.29	22*	118.38	18	101.78	30*	127.56	8*
Lumpy skin disease	118.59	15	116.80	14	121.30	15	110.34	24*	125.93	9*
LPAI	128.80	7	113.86	18*	137.25	2*	144.22	2*	119.87	17*
Monieziasis/taeniasis	129.35	5	114.38	17*	128.93	12*	144.45	1*	129.64	7
Nematodiasis	120.25	12	117.42	13	125.87	14	127.91	10	109.80	23*
New Castle disease	114.64	20	111.39	20	134.51	6*	114.68	19	97.98	32*
Paramphistomosis	98.93	35	111.38	21*	98.73	35	86.63	38*	98.98	31*
Pasteurellosis	118.62	14	113.43	19*	109.49	24*	121.31	14	130.26	6*

S6 Table. Continued

	Complete model		Reduced model							
			Expert group 1		Expert group 2		Expert group 3		Expert group 4	
Maladies	Mean score	Rank	Mean score	Rank	Mean score	Rank	Mean score	Rank	Mean score	Rank
PPR	114.31	21	129.05	6*	129.40	10*	95.42	33*	103.36	25*
RVF	130.74	3	138.13	2	135.36	4	119.12	15*	130.35	5
Q fever	108.05	25	115.47	16*	103.22	30*	113.47	21*	100.03	30*
Salmonellosis	120.75	11	128.13	7*	120.75	16*	112.94	23*	121.17	14*
Scabies	111.26	23	103.74	28*	126.34	13*	89.14	36*	125.83	10*
Trypanosomiasis	110.09	24	108.83	23	103.20	31*	125.36	11*	102.96	26
Tuberculosis	129.23	6	129.79	5	135.39	3*	118.33	16*	133.40	4
Viral hepatitis E	129.61	4	141.40	1*	134.07	7*	131.24	6	111.74	21*
Bivariate Spearman rank correlation between the complete model and reduced models (Rho correlation coefficient)			0.83		0.83		0.77		0.76	
p			<0.05		<0.05		<0.05		<0.05	

*Ranking changed three positions or more. ASF: african swine fever; BVD: bovine viral disease; CBPP: contagious bovine pleuropneumonia; CCPP: contagious caprine pleuropneumonia; CSF: classical swine fever; FMD: foot and mouth disease; HPAI: high pathogenic avian influenza; IBD; infectious bursal disease, IBR: infectious bovine rhinotracheitis, LPAI: low pathogenic avian influenza; PPR: small ruminant plague, RVF: Rift valley fever
