# Supplementary material for: Parallel multi-criteria decision analysis for sub-national prioritization of zoonoses and animal diseases in Africa: The case of Cameroon
Source: PLoS One. 2024 Jun 25;19(6):e0295742. doi: 10.1371/journal.pone.0295742 (PMC11198839; doi:10.1371/journal.pone.0295742)
Supplement: Attachment — Submitted filename: NoteOfRevision.docx [file pone.0295742.s014.docx]

**Note of revision**

Dear Editor,

Thank you for your email about our manuscript [PONE-D-23-22715]. We are grateful that our article falls within the scope of your journal, and we appreciate reviewers’ contributions and critical analyses of to improve the quality of our paper.

**Comments and suggestions for authors**

**comments**

**Comment 1:** Table 2. The text indicates Table 2 is how prioritization criteria were determined, but this title indicates these are the criteria. This must be clarified with more clear titles and descriptive text

**Response 1:** Table 2 indicates the criteria used to generate the list of diseases that were latter on ranked. Title changed to "Inclusion and exclusion criteria used to identify zoonoses and animal diseases to be prioritized".

**Comment 2:** How is this qualified? Published work?

**Response 2:** Part of the sentence "published and unpublished studies" was added.

**Comment 3:** Time frame for being present?

**Response 3:** Part of the sentence "during the study period" was added.

**Comment 4:** Are d and e different? If the only difference is the level of impact (significant vs any) that should be clarified

**Response 4:** In fact, “d” and “e” are different: socio-ecological versus socio-economic.

**Comment 5:** Line 141 This table doesn't appear to apply to this sentence - it appears Table 2 is creating a second list of diseases, no criteria.

**Response 5:** Deleted.

**Comment 6:** Line 172-174 Table 2 appears to be the inclusion exclusion criteria of diseases, not the prioritization criteria.

**Response 6:** The sentence was deleted.

**Comment 7:** Height

**Response 7:** Line 254 “Eight” was corrected.

**Comment 8:** Line 259. typo of eight, but should be seven I believe.

**Response 8:** Thank you. It is now corrected.

**Comment 9:** Line 260-262 sums to 7, not 8

**Response 9:** In fact, eight animal diseases (five viral, two bacterial, and one parasitic diseases). High pathogenic avian influenza was added.

**Comment 10:** Line 276, 277, 278, 280, 286, 288, 290, 291 (rewording).

**Response 10:** Corrected as suggested.

**Comment 11:** Table 6. I don't believe COVID-19 is considered primarily a zoonotic disease - if including it, there should be some discussion of how Figure 1 does not clarify the primary route of transmission for a disease, and therefore a disease with non-zoonotic primary transmission may still be included in the disease list. This should possibly also be part of the discussion - how does it impact result interpretation; is this a limitation within this work (why or why not).

**Response 11:** This was discussed in lines 389-397. Also read 43.Mackenzie, J.S.; Smith, D.W. COVID-19: a novel zoonotic disease caused by a coronavirus from China: what we know and what we don’t? Microbiol. Aust. 2020, 41(1), 45. <https://doi.org/10.1071/MA20013>.

**Comment 12:** Table 7, typo error.

**Response 12:** Q-Fièvre changed to "Q-fever".

**Comment 13:** Line 310: disagree with this use of the word modeling here. Suggest rewording to avoid the absolute implied here

**Response 13:** Changed to “MCDA methods are objective tools…”.

**Comment 14:** Line 331-332 awkward wording

**Response 14:** Changed to “There are several reasons to prioritize zoonoses and animal diseases in parallel”.

**Comment 15:** Line 346, 348 (rewording).

**Response 15:** Corrected as suggested.

**Comment 16:** Line 380-381 (rewording).

**Response 17:** Corrected as suggested.

**Comment 18:** Have all of these experts consented to having their names and contact information published in this paper?

**Response 18:** Informed consent was granted by all experts.
