# Supplementary material for: Parallel multi-criteria decision analysis for sub-national prioritization of zoonoses and animal diseases in Africa: The case of Cameroon
Source: PLoS One. 2024 Jun 25;19(6):e0295742. doi: 10.1371/journal.pone.0295742 (PMC11198839; doi:10.1371/journal.pone.0295742)
Supplement: Attachment — Submitted filename: Response To Reviewers.docx [file pone.0295742.s015.docx]

**RESPONSE TO REVIEWERS**

Dear Editor,

Thank you for your email about our manuscript [PONE-D-23-22715]- [EMID:7af7abfb763bdaab]. We appreciate reviewers and editor’s comments to improve the quality of our paper in order to meet PLOS ONE’s publication criteria.

**Journal requirements.**

Reference list reviewed. The list is complete and correct

**comments and responses**

**Comment 1**. Authors should correct all typographical and other errors in the manuscript e.g., SSA (Line 65) should be written in full at first use (Line 49); Pubmed should be changed to PubMed, Plos One to PLOS ONE, African journal online to African Journals Online (Line 118-119); valley to Valley (Line 263 and 311), and african to African (Line 309); Table 4 - New Castle to Newcastle (No. 28); valley to Valley (No. 29).

**Response 1.** Changes were made as requested.

**Comment 2**. Response to Comment 6: The phrase highlighted in yellow (line 172-174) by the reviewer was not deleted. Authors should see the phrase “...set, finally resulted in the identification of the most discriminating criteria that were therefore used as disease prioritization criteria.” (Line 173-175) in the revised manuscript and make necessary corrections.

Note that now Table 4 becomes Table 5.

**Response 2.** The reviewer’s comment was: « Table 2 appears to be the inclusion exclusion criteria of diseases, not the prioritization criteria”. Thus, we were supposed to give the correct table number for the phrase and not delete the phrase highlighted in yellow. Therefore we deleted “(Table 2)” for the previous reviewed manuscript and now replaced with “(Table 4)” which is the appropriate table number for the phrase.
